# Biomechanical Factors in the Adaptations of Insect Tibia Cuticle

**DOI:** 10.1371/journal.pone.0159262

**Published:** 2016-08-03

**Authors:** Eoin Parle, Hannah Larmon, David Taylor

**Affiliations:** Department of Mechanical and Manufacturing Engineering, Trinity Centre for Bioengineering, Trinity College, Dublin 2, Ireland; Natural Resources Canada, CANADA

## Abstract

Insects are among the most diverse groups of animals on Earth. Their cuticle exoskeletons vary greatly in terms of size and shape, and are subjected to different applied forces during daily activities. We investigated the biomechanics of the tibiae of three different insect species: the desert locust (*Schistocerca gregaria*), American cockroach (*Periplaneta americana*) and Death’s Head cockroach (*Blaberus discoidalis*). In a previous work, we showed that these tibiae vary not only in geometry (length, radius and thickness) but also in material quality (Young’s modulus) and in the applied stress required to cause failure when loaded in bending. In the present work we used kinematic data from the literature to estimate the forces and stresses arising *in vivo* for various different activities, and thus calculated factors of safety defined as the ratio between the failure stress and the *in vivo* stress, adjusting the failure stress to a lower value to allow for fatigue failure in the case of frequently repeated activities. Factors of safety were found to vary considerably, being as little as 1.7 for the most strenuous activities, such as jumping or escaping from tight spaces. Our results show that these limbs have evolved to the point where they are close to optimal, and that instantaneous failure during high-stress activities is more critical than long-term fatigue failure. This work contributes to the discussion on how form and material properties have evolved in response to the mechanical functions of the same body part in different insects.

## Introduction

Many natural materials exhibit mechanical properties that reflect their organism’s way of life. Morphological and material properties of natural structures can vary depending on the forces they experience or the uses to which they are put. Pine trees from windy or sheltered areas consist of the same material, but different geometries lend a stronger bending resistance to those exposed to windier conditions [1]. Studies carried out on the pleopods (forked swimming limbs) of isopod crustaceans [2] showed that the measured bending stiffness of the first 2 pairs of pleopods (used for providing thrust when swimming) was an order of magnitude larger than the 4th and 5th pairs (used primarily for respiration). The anterior (front) pairs had thicker cuticles, and a modulus of elasticity 12.5 times greater than the rearmost pair. This is an example of very similar structures on the same animal having significantly different geometries and material properties, both fit for their own individual purpose.

In a similar way, natural selection strives to optimize the skeleton and bone size of each animal species. Bones of increased thickness can endure higher forces, but suffer an increase in weight. Taylor and Dirks (2012) found that the radius to thickness ratio in locust hind tibia was close to optimal to resist the bending stresses applied during jumping with the best possible strength to weight ratio [3].

In engineering terms, a factor of safety defines the ratio of a structure’s failure strength to the maximum applied stress which it experiences in use. Depending on how the structure is designed, used, examined and maintained, the safety factor can range from as little as 1.5 in, for example, aircraft fuselages and pressure vessels [4] to more than 100 in, for example, safety critical components in power stations. Mammalian bone has a factor of safety for static loads as low as 2–4 [5–6]. For long-term fatigue failure, the safety factor for mammalian bone is actually less than 1.0, failure being prevented by continuous self-repair [7]. Apart from the case of mammalian bone, there has been little work done to investigate safety factors in biological structures: only a few researchers have addressed this matter for arthropod exoskeletons [8–10].

Insect legs are loaded during terrestrial locomotion (running and walking) and, in some species, during other activities such as jumping, grasping, digging, and swimming. These activities can be categorised as either “normal behaviour” such as walking and running, or “emergency behaviour”, which includes wedging (pushing through a small hole or crevice), righting (when overturned) and jumping to escape predators, or to take flight. Normal behaviour generally involves smaller forces but more repetitions, raising the possibility of long term material failure by fatigue. The kinematics of many of these activities has been previously researched in some detail [3], [11–15].

The present work brings together the results of kinematic studies, measurements of limb geometry and measurements of cuticle material properties. By analysing these various results we were able to estimate safety factors for a range of activities in three different insects.

## Materials & Methods

### Insects

In two previous papers [16, 17] we measured the dimensions and mechanical properties of four different tibiae from three insect species: the metathoracic (hind) tibiae of the American cockroach (*Periplaneta americana*), the Deaths Head cockroach (*Blaberus discoidalis*) and the desert locust (*Schistocerca gregaria*) and also the mesothoracic (middle) tibia of the desert locust. These are all orthopteroid species, so are relatively closely related, but the three differ in size, shape, weight and daily activities, and as such, the stresses experienced by their tibiae *in vivo* were expected to differ significantly also. Insects were sourced from Blades Biological Ltd (Cowden, Edenbridge, Kent, TN8 7DX, UK). All insects tested were adults 2–3 weeks post final moult. Insects from individual species were of the same sex with the exception of the Death’s Head cockroach, where no single sex could be sourced from our supplier. All animals were reared in a 12hr light / 12hr darkness environment and fed dry cereals and leafy vegetables *ad libitum*. All tibiae were removed directly from live insects after sedation by cutting just below the tibia-femoral joint (the knee). Specimens were tested within 10 minutes of removal to minimize the effect of desiccation [17]. All experiments were performed in accordance with the Animals (Scientific Procedures) Act of 1986, and no endangered species were used.

### Experimental procedure

**Fig 1:**
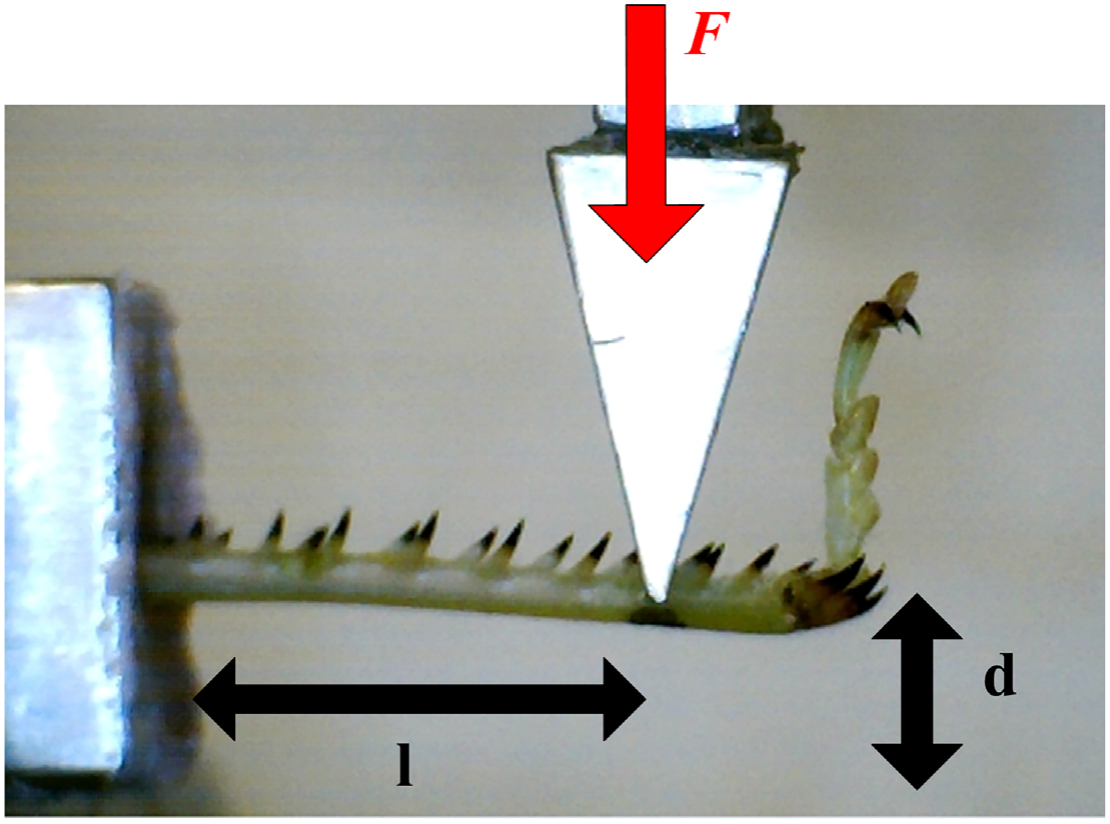
Experimental rig. Photograph showing cantilever length (*l*), tibia deformation (*d*) and applied force (*F*).

All mechanical tests were performed using a tensile / compression testing machine (Zwick / Roell Z005, Ulm, Germany), fitted with a 5 Newton load cell, and the testing rig shown in Fig 1. Immediately after removal, legs were set in a well of polymer cement (Howmedica^®^ Surgical Simplex P Bone Cement mixed at a 1:1 ratio for rapid hardening). Once set, a cantilever load was applied in the dorsal-ventral plane. The cantilever length (*l*) was measured, and readings for applied force (*F*) and displacement (*d*) were recorded: displacement was gradually increased at a rate of 5 mm/min until failure occurred. After testing, the samples were stored in a 3.7% glutaraldehyde fixative for 24 hours, after which they were preserved in 70% ethanol solution.

### Measurements and calculations

Whole body weight was obtained for insects before testing. A scanning electron microscope (SEM, Zeiss Ultra Plus, Oberkochen, Germany) was used to measure tibia dimensions after testing. Several slices were taken from each tibia. 10–20 measurements of tibia diameter and thickness were taken from each slice using Fiji image analysis software (an Open Source Image Processing package based on ImageJ). Final thickness and radius values used for each tibia were averages of these values. Cross sectional area was calculated using:

$$A=\;\pi (r^{2}-{\left(r-t\right)}^{2})$$ (1)

Where *r* = tibia radius and *t* = cuticle thickness.

For the purpose of calculating stress and strain, the tibiae were assumed to be hollow tubes of circular cross section, and the cuticle material was assumed to be the same throughout. This followed the approach taken in our previous work [3], [16–19]. In practice some tibiae vary from being perfectly circular, having an elliptical shape, and the cuticle is known to consist of several layers, especially the exocuticle and endocuticle, which have different properties [20].

Given these assumptions, the bending stress (σ) experienced by the sample can be calculated using the flexure formula:

$$\sigma =\;\frac{Flr}{I}$$ (2)

Where *F* = applied force, *l* = cantilever length (distance from applied force to fixation point, see Fig 1) and *I* = moment of inertia, calculated using:

$$I=\;\frac{\pi }{4}\left(r^{4}-{\left(r-t\right)}^{4}\right)$$ (3)

The strain experienced by the sample is given by:

$$\varepsilon =\;\frac{3dr}{l^{2}}$$ (4)

Where *d* = sample deflection.

The material stiffness (Young’s modulus) was estimated from the slope of the linear portion of the loading curve (stress v strain). The above equations assume that the length is much larger than the radius (lr>20, [21]). With some of the smaller specimens, this condition was not met. To correct for this geometrical deviance, individual computer models were made of some of these specimens using finite element modelling. Comparing the stresses obtained from the computer model with those predicted by the equation allowed us to correct the stress estimates for the legs with low lr values. Inputting the experimental Young’s Modulus to the model, and comparing experimental and modelled deflections gave a correction factor for the Young’s. The values for stress and stiffness in the Results section include these corrections.

### Statistics

Statistical tests were performed using IBM’s SPSS (Version 19). Univariate ANOVA tests were performed with Tukey-Kramer HSD (for unequal sample sizes) *post-hoc* tests to analyse significance between tibia groups with regards to strength and stiffness.

### Biomechanics

Ground reaction forces experienced *in vivo* were taken from previous literature (See Table 1), and used to calculate stresses induced in each tibia for various activities.

**Table 1:** Previously measured values of ground reaction forces for different insects. F_Z_ and F_X_ denote the vertical and horizontal force components respectively and F_R_ denotes the resultant of the two.

*Insect*	Activity	Ground Reaction Force (N)
*P*. *americana* [11]	Running	0.015 (F_Z_) 0.003 (F_X_)
*B*. *discoidalis* [12]	Walking	0.011 (F_Z_) 0.005 (F_X_)
*F*. *polyctena* [15]	Walking	0.070 (F_R_)
*B*. *discoidalis* [13]	Righting	0.142 (F_Z_) 0.034 (F_X_)
*B*. *discoidalis* [14]	Wedging	0.18 (F_R_)
*S*. *gregaria* [22–23]	Jumping	0.15 (F_R_)

Full and Tu (1991) experimentally determined the force on the American cockroach hind-leg while *running* at 0.92 m/s [11]. This was scaled up by 1.38 to compensate for the larger average weight of our cockroaches. Full, Blickhan *et al*. (1991) determined the force on the Death’s Head cockroach hind-leg while *walking* at 0.38 m/s [12].

No published data currently exists for the forces experienced by the locust mid-leg during walking. A value was estimated using two sets of data: the Death’s Head cockroach mid-leg [12] and the ant (*Formica polyctena*) mid leg [15], scaling appropriately to allow for the difference in body weight. All three insects display the classic “tripod” walking gait. The resultant force values from these estimations were 12.9 mN (scaling from ant) and 7.8 mN (scaling from Death’s Head cockroach). These two estimates are relatively similar given the uncertainties involved: an average of the two was used.

Data from Full and Ahn (1995) and Full, Yamauchi *et al*. (1995) [13–14] was used for emergency activities of “wedging” and “righting” respectively for the Death’s Head cockroach (Table 1). A cockroach can flatten its body to squeeze through tight crevices less than half its body height [24] due to soft connecting membranes between the stiff sclerites covering its body. When a cockroach squeezes or “wedges” through a small slot, it thrusts its hind-legs alternately, which can produce a ground reaction force four times greater than body weight. Wedging and righting give a better indication than running of the maximum capacity of a single leg to generate force. Forces experienced during righting were up to 8 times higher than those experienced during running, and were even greater for wedging.

**Fig 2:**
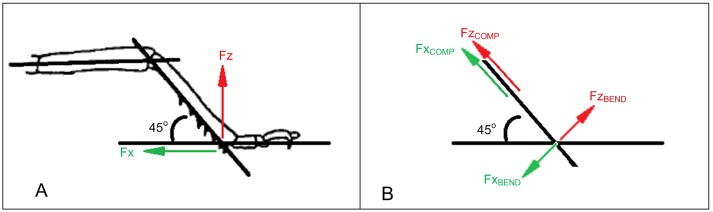
Resolving forces. Diagram showing force resolution from horizontal and vertical “ground reaction forces” into bending and compression forces.

In our cantilever bending tests, forces were applied perpendicular to the tibia. Ground reaction forces in all previous literature mentioned above are expressed in terms of F_X_ and F_Z_: horizontal and vertical components of the force for an insect traversing a smooth level surface, as pictured in Fig 2(a). Not pictured is a lateral force, F_Y_ also expressed in the literature and accounted for in our calculations. The ground reaction forces were resolved into pure compression (force component parallel to the tibia) and pure bending (force component perpendicular to the tibia) as shown in Fig 2(b).

**Fig 3:**
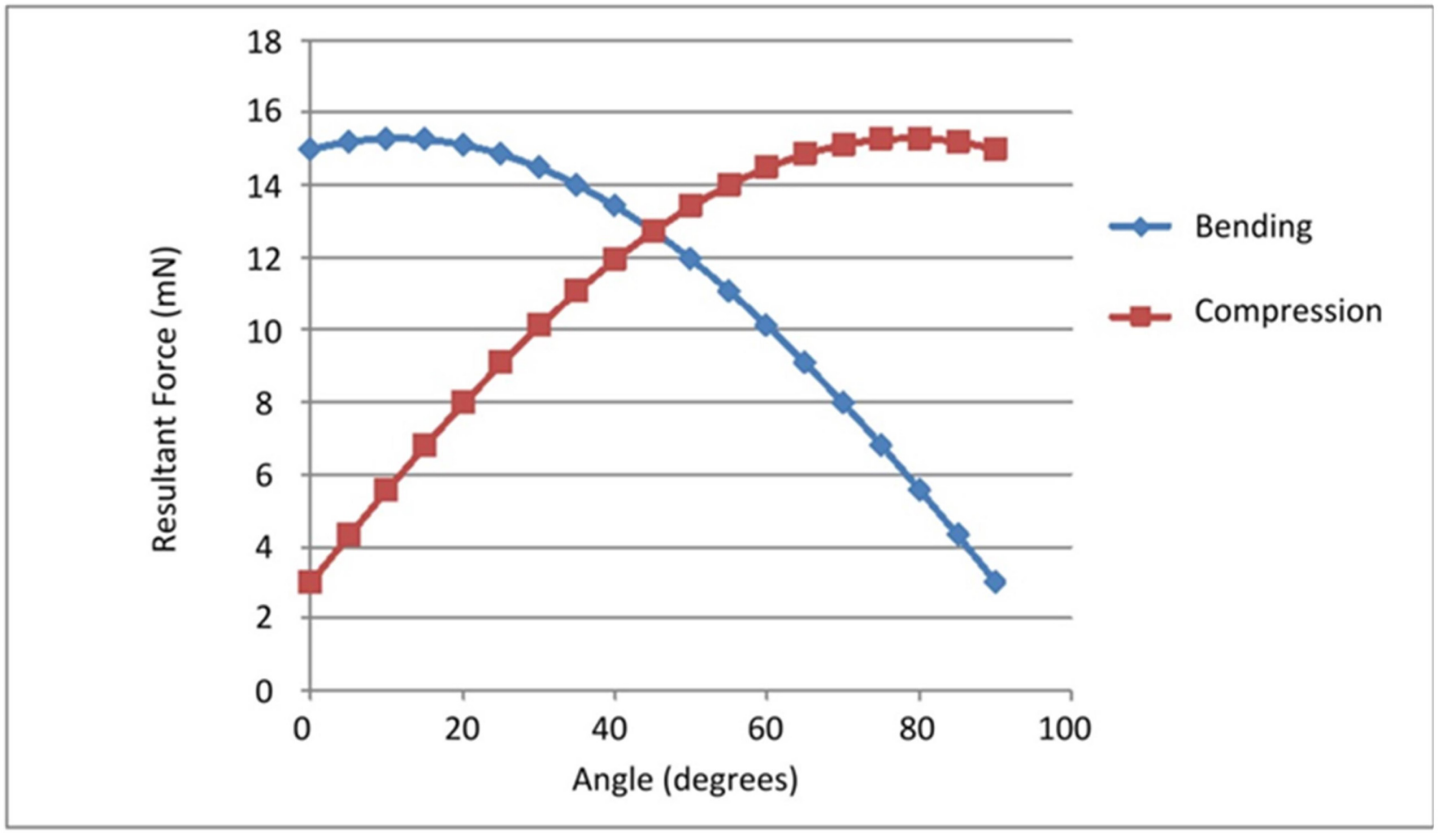
Force dependant on angle. Graph showing how bending and compressive forces differ based on the angle of the tibia relative to the ground. Data plotted are resultant forces for the running *P*. *americana* with F_Z_ = 15 mN, F_X_ = 3 mN.

During gait, the angle between the leg and the ground varies. The angle of peak force experienced for various tibiae can vary widely [25–26]. In our calculations we assumed an angle of 45° as a typical value. As the example in Fig 3 shows, the bending contribution of the force is relatively constant for all angles up to 45°, so our results are not strongly affected by this assumption.

Having estimated the resultant bending force, the *in vivo* bending stress was calculated using the flexure formula (Eq 2), and average tibia dimensions for each species. We found that the stresses generated by the axial compressive loads were negligible compared to the bending stresses. Where necessary, the forces applied during walking were scaled up by a factor of 3 to estimate running stresses, and *vice versa*. This is a common approach in mammalian biomechanics—forces experienced while running are typically three times greater than those experienced while walking [27]. Although insects differ in their gait kinematics from mammals, their tripod gait has been described as a running or bouncing gait [28], similar to that of trotting quadrupeds or running bipeds [29–30].

## Results

**Fig 4:**
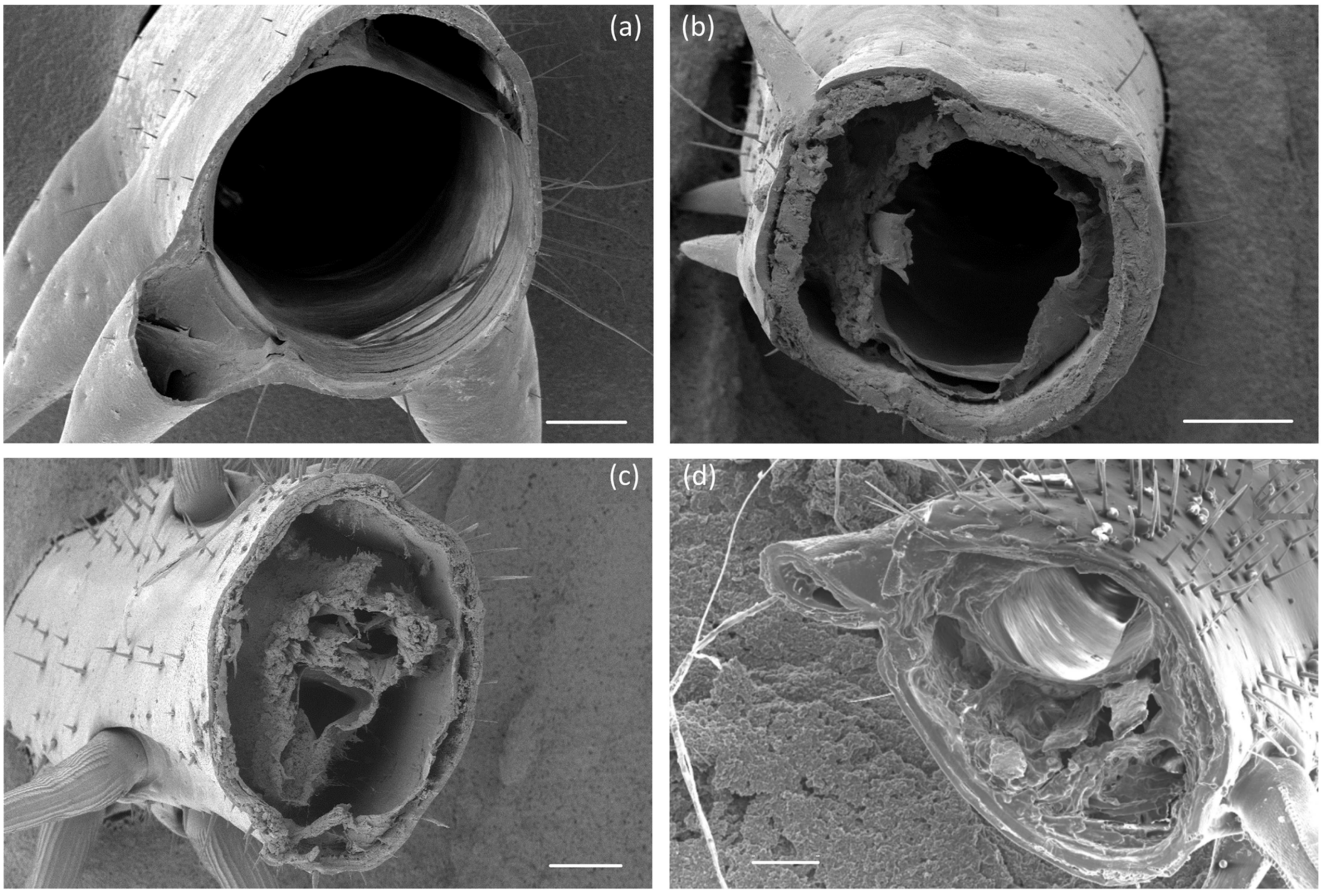
Cross section of legs examined. SEM images showing cross sections of the locust hind-leg (a), locust mid-leg (b), American Cockroach hind-leg (c) and Death’s Head Cockroach hind-leg (d). All scale bars measure 200 μm.

Fig 4 shows typical images of the cross sections of the four tibia types. Table 2 shows measured values of tibia dimensions and insect weight. There was relatively little variability (i.e. standard deviation) in dimensions within species for the tibiae examined. The locust mid-leg, though small in terms of length and radius, was found to be relatively thick-walled. The two locust tibiae had almost identical cross sectional areas despite having different radius and thickness values. Differences in insect weight for the three species are reflected in differences in the cross sectional area of the tibia.

**Table 2:** Relevant dimensions (mean values ± one standard deviation) for locust mid-legs (n = 26), American cockroach (n = 17) and Death’s Head cockroach (n = 10), and insect total weight (n = 10 for all specimens). Data for locust hind-legs in all tables is from Dirks and Taylor (2012) [17].

	Radius (mm)	Thickness (mm)	Area (mm^2^)	Length (mm)	Insect Weight (g)
Locust Hind-Leg	0.594 ± 0.04	0.054 ± 0.007	0.192	20.3 ± 4.8	1.55 ± 0.28
Locust Mid-leg	0.403 ± 0.04	0.086 ± 0.01	0.194	4.94 ± 0.3	1.55 ± 0.28
American Cockroach	0.409 ± 0.05	0.063 ± 0.02	0.149	5.98 ± 0.8	1.12 ± 0.21
Death’s Head Cockroach	0.574 ± 0.06	0.076 ± 0.02	0.256	8.34 ± 1.3	3.36 ± 0.48

### Mechanical properties

**Fig 5:**
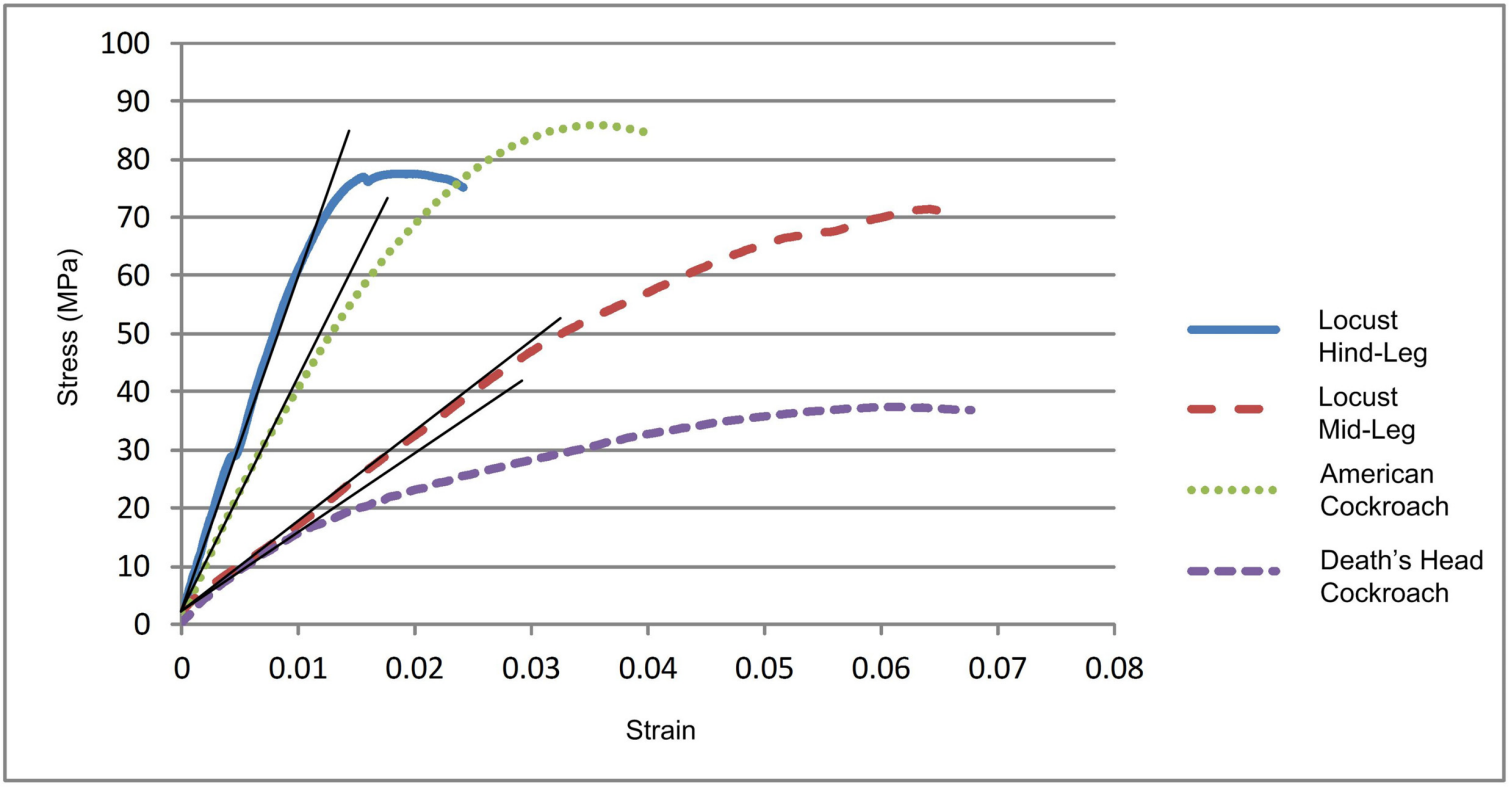
Loading curves. Stress/strain data for a typical sample of each insect tibia. Shown is the initial linear slope of the loading curve which was used to estimate Young’s Modulus (E). The maximum stress for each test was taken to be the failure strength of the tibia.

**Fig 6:**
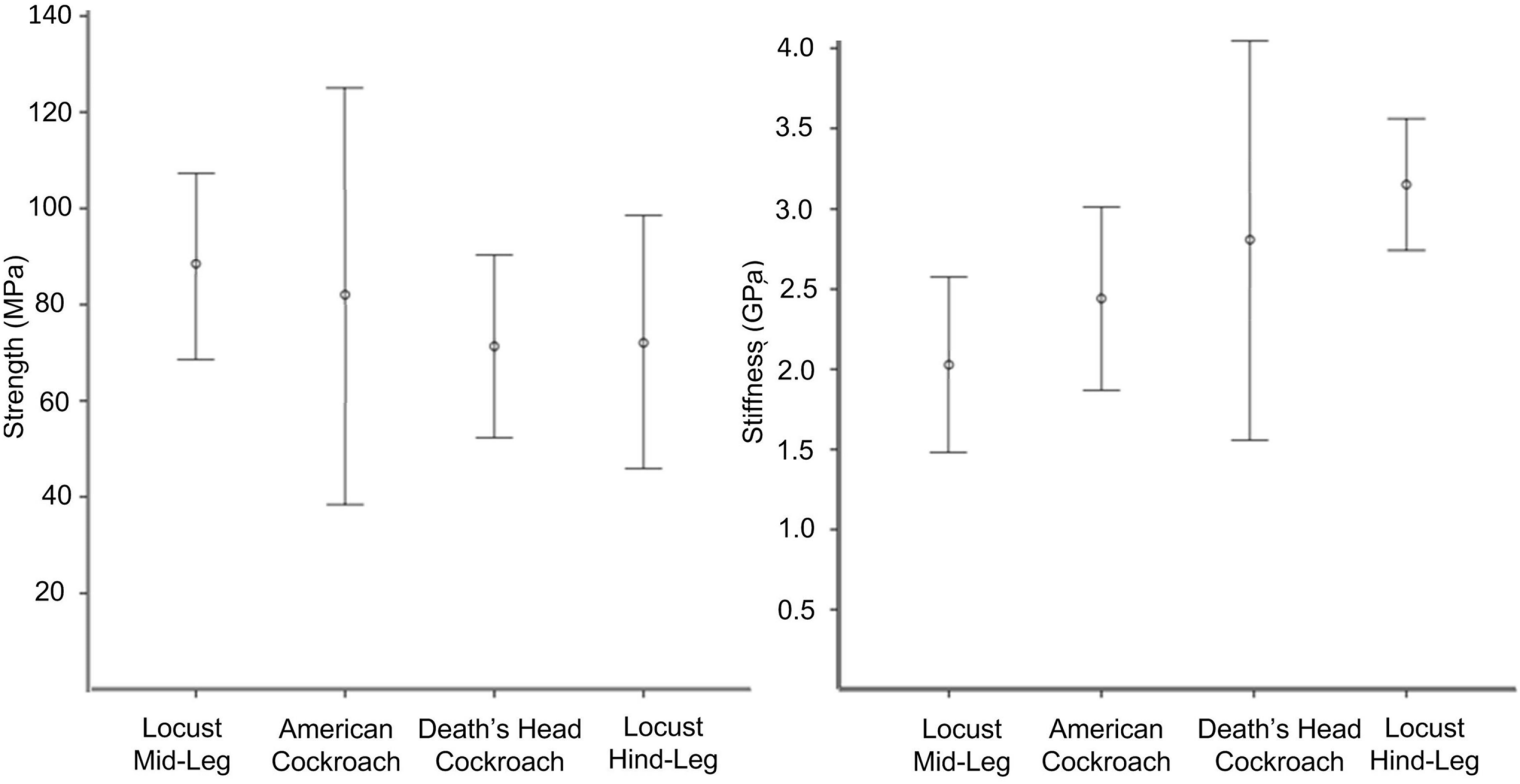
Mechanical properties. Experimentally measured mechanical properties with error bars showing one standard deviation.

Fig 5 shows typical stress/strain curves for the four types of tibia. Cuticle material stiffness (Young’s Modulus) was obtained from the slope of the linear portion of the curve. Some samples behaved in a non-Hookean manner (i.e. displayed a non-linear loading curve). For these, the initial tangent modulus was used to estimate Young’s Modulus. Failure strength was defined as the maximum stress endured during a test: results are summarised in Table 3 and Fig 6.

**Table 3:** Experimentally measured mechanical properties. Shown are mean values ± 1 standard deviation.

	Young’s Modulus (GPa)	Failure Stress (MPa)	Failure Strain
Locust Hind-Leg	3.05 ± 0.6	72.05 ± 30.5	-
Locust Mid-leg	1.95 ± 0.65	88.51 ± 19.3	0.055 ± 0.02
American Cockroach	2.34 ± 0.72	82.06 ± 45.5	0.040 ± 0.02
Death’s Head	2.72 ± 1.46	72.9 ± 19.8	0.036 ± 0.01

Initial univariate ANOVA analysis showed that the variables insect species and leg type (hind or middle) significantly affected the Young’s modulus but not the failure strength of the tibiae. While failure strength did not differ significantly (P = 0.316) among species, *post-hoc* Tukey-Kramer HSD analysis (for unequal sample sizes) showed that the Young’s modulus of the locust hind-leg cuticle differed significantly from that of the locust mid-leg (P < 0.0001) and the American cockroach hind leg (P < 0.01). No other difference in stiffness between the tibia types was significant (P > 0.05). This variation in the material’s Young’s Modulus indicates that different materials are present in these tibiae.

### Biomechanics

Table 4 shows calculated bending stresses for the different species during various activities. For the three cases of emergency behaviour for which data were available, jumping in the locust hind leg was found to generate the greatest amount of stress. Wedging and righting activities in the Death’s Head cockroach gave rise to significantly higher stresses than normal walking and running activities.

**Table 4:** *In vivo* stresses experienced by the tibia of each insect when walking, running, and during emergency behaviour (jumping, wedging and righting). Underlined numbers are direct calculations based on data from the literature. Figures in italics are estimates achieved by scaling up or down from force data in the literature.

	Walking Stress (MPa)	Running Stress (MPa)	Emergency Behaviour Stress (MPa)
Locust Mid-Leg	*2*.*15*	*6*.*45*	-
American Cockroach	*1*.*96*	5.87	-
Death’s Head Cockroach	1.89	*5*.*67*	-
Locust Hind-Leg (jumping)	-	-	42.2
Death’s Head Cockroach (wedging)	-	-	23.29
Death’s Head Cockroach (righting)	-	-	18.89

### Factor of safety

Table 5 shows estimates of the safety factor (failure stress divided by *in* vivo stress) for each tibia under various loading conditions. An insect tibia will be subjected to a large number of repeated cycles when walking and running, which may lead to a fatigue failure. Locusts have been recorded walking constantly for long periods of time in laboratory conditions [26]. They gradually build up their pace to a high intensity which can be sustained for up to 2 hours. The highest recorded stride frequency was 8 Hz, implying that almost 58,000 continuous cycles at a time is a regular occurrence for a locust. One can assume that the cockroaches would experience similar or greater numbers of sustained cycles based on their average stride frequencies of 14 Hz (Death’s Head) and 25 Hz (American). Previously, we found that the stress to cause fatigue failure after a large number of cycles (10^5^) in the locust hind tibia is approximately half the static failure strength [18]. So this factor has been applied in calculating the relevant stress to failure and applied to the running stresses in Table 5. We assumed that emergency behaviour (jumping, wedging, righting) would not be repeated often enough for fatigue failure to be an issue. Bennet-Clark (1975) showed that the locust cannot perform more than 10–20 jumps consecutively [22] (due to limitations of the muscles, not the cuticle), and the cockroach’s wedging and righting actions are performed only infrequently [13–14]. Therefore it can be assumed that the static strength is the relevant stress to cause failure in these cases.

**Table 5:** Safety factors of insect tibiae for various situations. Relevant stress to cause failure is divided by *in vivo* applied stress to obtain the safety factor.

	Relevant stress to cause failure (MPa)	*In vivo* applied stress (MPa)	Safety Factor
Locust Mid-Leg (*running*)	44.25	6.45	6.8
American Cockroach (*running*)	41.03	5.87	7.0
Death’s Head (*running*)	36.45	5.67	6.3
Locust Hind-Leg (*jumping*)	72.05	42.20	1.7
Death’s Head (*wedging*)	72.90	23.29	3.1
Death’s Head (*righting*)	72.90	18.89	3.9

## Discussion

This work represents the first systematic study to compare dimensions, mechanical properties and *in vivo* stresses in order to define factors of safety for the same body part in several different insects, carrying out different activities. The dimensions of the tibiae vary significantly, which is not surprising considering the different insect weights and different purposes to which the limbs are put. Less obviously, the cuticle material itself was also found to be different, having different Young’s modulus values in all four cases. This implies differences in chitin fibre amount and orientation, in the degree of hardening by sclerotization, or in the relative amounts of exocuticle and endocuticle.

Though Young’s modulus varied considerably, strength (defined as the maximum bending stress to cause failure of the tibia) did not significantly vary from one species to another. This apparent contradiction is explained by the fact that these limbs failed by buckling, as was established in previous work [16], where it was shown that buckling occurs before the material reaches its tensile strength.

This analysis has some limitations: a simplified shape (hollow circular cylinder) was assumed for the cross section, and linear elastic material behaviour was assumed. In practice, the stress-strain curves (Fig 5) always showed a non-linear region before maximum stress, suggesting inelastic behaviour in the material, such as viscoelasticity, plastic deformation or sub-critical damage. However, this area of non-linearity does not interfere with our assumptions, as the safety factors calculated for each insect imply that they are usually operating within the linear region of the stress-strain curve.

The locust mid-leg and hind-leg have similar cross-sectional areas, but the locust hind-leg is relatively thin-walled (larger radius, smaller thickness). Although both have similar failure strengths, the bending moment required to cause failure is significantly higher (by 35%) for the hind-leg (3.76 Nmm) than the mid-leg (2.79 Nmm). The hind-leg experiences greater bending moments *in vivo*, and is enabled to do so by the superior material (as evidenced by the higher Young’s Modulus). This indicates that cuticle composition and geometry can vary from leg to leg on a single insect to best resist the forces experienced *in vivo*.

The two cockroaches show some distinct physical differences. The Death’s Head cockroach is much larger and heavier than the American (3.36 g compared to 1.12 g). This is reflected in the tibia geometries—the Death’s Head tibia having a larger radius and thickness, giving a 50% larger cross-sectional area. One might expect material properties such as strength and stiffness to scale as a function of the insect’s weight, but this is not the case. Although three times the weight of the American cockroach, the Death’s Head cockroach hind-tibia displays no significant difference in strength or stiffness. Why then does an insect which is so much smaller and lighter require material of the same strength and quality as a much larger one? Both insects experience very similar stresses when running and when walking. Both cockroaches display a similar walking gait, and their patterns of ground reaction force are described as being “not qualitatively different” [11] even when the American cockroach abandons the tripod gait when running.

The Death’s Head cockroach operates over a smaller range of speeds. Its stride frequency increases from 3–13 Hz as its speed increases. Once it reaches a max of 13 Hz, increased stride length is used to increase its speed [31]. *Periplaneta* shows relatively little change in stride frequency (20–25 Hz) over a large range of speeds—stride length accounts for most of the increase in speed. It operates at frequencies close to wingbeat frequencies during flight [31]. This illustrates that the smaller American cockroach favours running, and can do so at maximum speeds of up to 1.0–1.5 m/s (or 50 body lengths per second [11], producing greater forces (and greater strain rates) in proportion to its body weight compared to that of the Death’s Head cockroach. Maximum speeds for the Death’s Head are less than half that found for the American. Also, the Death’s Head is a more awkward runner [11], wasting more energy pitching and rolling than the American. This explains why the stresses experienced by these two insects’ hind tibiae are so similar, and thus why they are so closely matched in terms of strength and material stiffness.

Our biomechanics analysis has revealed safety factors which in some cases are very low, as small as 1.7 and 3.1, comparable to some of the smallest safety factors found in engineering structures. The limb segments of these insects have evolved to operate at applied stresses which are very close to their limiting values. Even allowing for the possibility of fatigue failure in common activities such as running, in which the leg is loaded many times, it emerged that the lowest safety factors occurred during strenuous activities which are performed only occasionally, such as jumping and wedging. Although force measurements for wedging in American cockroaches could not be found, they are known to squeeze through gaps as small as 4 mm [32] while still maintaining a relatively high speed. One could speculate that a similar safety factor applies for performing such activities.

The safety factor of 6.8 for the locust mid-leg during running is probably an underestimate because it assumed a high running speed of 0.92 m/s maintained for long periods: flying could take preference when long distances must be travelled. It certainly seems that this tibia is much stronger than it needs to be, a finding which requires further work to explain. Although during the tripod gait, the middle leg must bear double the weight of the front and hind leg on the opposite side, it has been shown that the hind-leg endures the highest forces, possibly providing more propulsion than the middle-leg. It may be possible that the mid-leg will also endure some emergency behaviour of its own (perhaps clinging to a surface during an attack or a windstorm), or may experience high forces during landing after jumping or flying. The mid leg also has to endure forces other than ground reaction forces that may influence its structure. Muscles in the locust front two leg pairs are continuously active even when the locust is stationary [31]. The applied force, though relatively small, could possibly occur more frequently *in vivo* than walking forces, which could be why they seem to be “distinctly over-built” [33]. Because of the specialization of its hind-leg for jumping, the locust also shows a very high variability in the stepping movement of its legs regarding the protraction / retraction phases (compared to the cockroach [34]), which could also lead to more unpredictable ground reaction forces for the mid-legs.

Other workers found relatively low safety factors in some arthropod body parts. Bennet-Clark (1975) estimated a safety factor of 1.2 for the extensor apodeme (tendon) of locust hind leg during jumping [22]. Palmer, Taylor *et al*. (1999) and Taylor, Palmer *et al*. (2000) estimated the safety factor for six species of predatory *Cancer* crabs (*Crustacea brachyura*) by comparing the exerted biting force to the breaking strength of their claws [8–9]. These varied within and among the species depending on stage of development and wear, and ranged from 2 to 7. McCullough (2014) found the safety factor for the horn of the rhinoceros beetle (*Trypoxylus dichotomus)* to range from 3.5 to 10.3, which scaled with the inverse of the insect size [10]. Some insect limbs contain specializations such as the longitudinal ridges on a stick insect leg, or the triangular cross-section of a bumblebee leg [16]. The locust hind-leg also contains a buckling region examined by Bayley et al., 2012 [35], which was excluded from this study. This may contribute to increasing the safety factor of the locust hind-leg further than the 1.7 we see in this study.

All safety factors are average values. Both the failure strength and the applied force have some variability associated with them. The measured mechanical properties had standard deviations between 20% and 50% of the mean values, which is considerably greater than typically found in engineering structures. The applied force will also vary considerably *in vivo*: currently there is no data on this variability. This implies that situations in which the failure force exceeds the limb strength will occur fairly often. A detailed probabilistic analysis, such as has been conducted for mammalian bones [36] would require more information and is beyond the scope of this study, however previous literature shows that failures do occur with quite high probabilities. Taylor, Palmer *et al*. (2000) noticed a breakage frequency in natural populations of roughly 6% across six species of crab [9], while McCullough (2014) observed that 17% of the population of Rhinoceros Beetle tested showed some sign of injury, while 4% had severely damaged horns from battles with rivals [10]. The limbs being tested in this study might be expected to evolve with rather high failure probabilities. This is the price of being light and economic in energy terms; sacrificing individual insects but making the species as a whole more viable. Further analysis along these lines would provide a very interesting topic for future research.

## Conclusions

Animals expend considerable resources on the construction of weight-bearing body parts. Failure must be avoided, within a reasonable probability, but using too much material is wasteful of food and energy and, by increasing weight, impedes motion. It is to be expected that evolution will operate to strike a balance between these competing factors. However, little is known about how this operates in practice in the bodies of insects and other arthropods. In the present work we have shown that the form and material properties of the tibiae in different insect species are governed by the need to resist occasional strenuous activities rather than frequent, regular activities, and that evolution has created body parts with relatively low safety factors, presenting significant risks of failure whilst ensuring a light, economic limb form.

## References

[1] Telewski FW and Jaffe MJ (1986). Thigmomorphogenesis: field and labratory studies of *Abies fraseri* in response to wind or mechanical perturbation. *Physiologia Plantarum* 66: 211–218. 11538654 10.1111/j.1399-3054.1986.tb02411.x

[2] Alexander DE, Blodig J and Hseih S-Y (1995). Relationship between function and mechanical properties of the pleopods of isopod crustaceans. *Invertebrate Biology* 114(No. 2): 169–179.

[3] Taylor D and Dirks J-H (2012). Shape optimization in exoskeletons and endoskeletons: a biomechanics analysis. *Journal for the Royal Society Interface* 9(77): 3480–3489 10.1098/rsif.2012.0567 PMC3481597 22977103

[4] Burr A and Cheatham J. Mechanical Design and Analysis, section 5.2, Prentice-Hall. 1995.

[5] Currey JD. Bones: Structure and Mechanics. Princeton NJ, Princeton University Press 2002.

[6] Sabick MB, Torry MR, Kim Y-K and Hawkins RJ (2004). Humeral torque in professional baseball pitchers. *American Journal of Sports Medicine* 32: 892–898 15150034 10.1177/0363546503259354

[7] Taylor D, Hazenberg JG and Lee TC (2007). Living with Cracks: Damage and Repair in Human Bone. *Nature Materials* 2: 263–268 10.1038/nmat1866 17401419

[8] Palmer RA, Taylor GM and Barton A (1999). Cuticle Strength and Size-Dependence of Safety Factors in Cancer Crab Claws. *The Biological Bulletin* 196: 281–294 28296491 10.2307/1542953

[9] Taylor GM, Palmer AR and Barton AC (2000). Variation in safety factors of claws within and among six species of Cancer crabs (Decapoda: Brachyura). *Biological Journal of the Linnean Society* 70: 37–62

[10] McCullough EL (2014). Mechanical limits to maximum weapon size in a giant rhinoceros beetle. Proceedings for the Royal Society Series B: Biological Sciences 281 (1786) 10.1098/rspb.2014.0696 PMC4046419 24827447

[11] Full RJ. and Tu MS (1991). Mechanics of a rapid running insect: two-, four- and six legged locomotion. *Journal of Experimental Biology* 156: 215–231 2051129 10.1242/jeb.156.1.215

[12] Full RJ, Blickhan R and Ting LH (1991). Leg design in hexapedal runners. *Journal of Experimental Biology* 158: 369–390 1919412 10.1242/jeb.158.1.369

[13] Full RJ, Yamauchi A and Jindrich DL (1995). Maximum single leg force production: cockroaches righting on photoelastic gelatin. *Journal of Experimental Biology* 198: 2441–2452 9320366 10.1242/jeb.198.12.2441

[14] Full RJ and Ahn AN (1995). Static forces and moments generated in the insect leg: comparison of a three-dimensional musculo-skeletal computer model with experimental measurements. *Journal of Experimental Biology* 198: 1285–1298 9319155 10.1242/jeb.198.6.1285

[15] Reinhardt L, Weihmann T and Blickhan R (2009). Dynamics and kinematics of ant locomotion: do wood ants climb on level surfaces? *Journal of Experimental Biology* 212(Pt 15): 2426–2435 10.1242/jeb.026880 19617436

[16] Parle E, Herbaj S, Sheils F, Larmon H and Taylor D (2015). Buckling failures in insect exoskeletons. *Bioinspiration and Biomimetics*. 11 016003. 26678374 10.1088/1748-3190/11/1/016003

[17] Dirks J-H. and Taylor D (2012). Fracture toughness of locust cuticle. *Journal of Experimental Biology* 215 (Pt 9): 1502–1508 10.1242/jeb.068221 22496286

[18] Dirks J-H., Parle E and Taylor D (2013). Fatigue of insect cuticle. *Journal of Experimental Biology* 216(Pt 10): 1924–1927 10.1242/jeb.083824 23393276

[19] Parle E and Taylor D (2013). The self-healing properties of insect cuticle. Journal of Postgraduate Research, Trinity College Dublin. Volume XII, 90–111

[20] Klocke D and Schmitz H (2011). Water as a major modulator of the mechanical properties of insect cuticle. *Acta Biomateriala* 7(7): 2935–2942 10.1016/j.actbio.2011.04.004 21515418

[21] Young WC (1989). Roark’s Formulas for Stress and Strain, International Edition, McGraw-Hill Book Company

[22] Bennet-Clark HC (1975). The energetics of the jump of the locust *Schistocerca gregaria*. *Journal of Experimental Biology* 63: 53–83 1159370 10.1242/jeb.63.1.53

[23] Alexander RM (1995) Leg design and jumping technique for humans, other vertebrates and insects. *Philosophical Transactions of the Royal Society of London B* 347, 235–248. 10.1098/rstb.1995.0024 7777591

[24] Jayaram K and Full RJ (2016). Cockroaches traverse crevices, crawl rapidly in confined spaces, and inspire a soft, legged robot. *Proceedings of the National Academy of Sciences of the United Stateds of America*. 11, 8 E950–E957, 10.1073/pnas.1514591113 PMC4776529 26858443

[25] Dudek DM, and Full RJ (2006). Passive mechanical properties of legs from running insects. *Journal of Experimental Biology* 209: 1502–1515. 16574808 10.1242/jeb.02146

[26] Moorehouse JE, Fosbrooke IHM and Kennedy JS (1978). "Paradoxical driving" of walking activity in locusts. *Journal of Experimental Biology* 72: 1–16

[27] Whalen RT, Carter DR and Steele CR (1988). Influence of physical activity on the regulation of bone density. *Journal of Biomechanics* 21: 825–837 3225269 10.1016/0021-9290(88)90015-2

[28] Full RJ and Tu MS (1990). Mechanics of six-legged runners. *Journal of Experimental Biology*, 148, 129–146 2307925 10.1242/jeb.148.1.129

[29] Cavagna GA, Saibene FP and Margaria R (1964). Mechanical work in running. *Journal of Applied Physiology* 19: 249–256 14155290 10.1152/jappl.1964.19.2.249

[30] Cavagna GA, Heglund NC and Taylor CR (1977). Mechanical work in terrestrial locomotion: two basic mechanisms for minimizing energy expenditure. *American Journal of Physiology* 233: 243–261 10.1152/ajpregu.1977.233.5.R243 411381

[31] Chapman RF. The insects: structure and function, Cambridge University Press (2013).

[32] Jayaram K, Springthorpe D, Haldane D, DiRocco A, McKinley S and Full R (2013). Challenges of confined space locomotion—a case study using the American cockroach A1.42, 6 July. Society of Experimental Biology Annual Meeting. Valencia, Spain

[33] Katz SL and Gosline JM (1992). Ontogenetic scaling and mechanical behaviour of the tibiae of the African desert locust (Schistocerca gregaria). *Journal of Experimental Biology* 168: 125–150

[34] Burns MD (1973). The control of walking in orthoptera I: leg movements in normal walking. *Journal of Experimental Biology* 58: 45–58

[35] Bayley TG, Sutton GP and Burrows M (2012) A buckling region in locust hindlegs contains resilin and absorbs energy when jumping or kicking goes wrong. *Journal of Experimental Biology* 215, 1151–1161. 10.1242/jeb.068080 22399660

[36] Taylor D (2011). What we can’t learn from nature. *Materials Science and Engineering*: *C* 31: 1160–1163.

